# The Treatment of Acne Vulgaris

**Published:** 1907-07-20

**Authors:** M. Stewart Smith


### The General Practitioner's Column.

[Contributions to this Column are invited, and if accepted will be paid for.]

### THE TREATMENT OF ACNE VULGARIS.

By M. STEWART SMITH, M.B., B.S.Lond., M.R.C.S.Eng.

In acne vulgaris the underlying state is one of seborrhœa, and this fact must be realised if satisfactory results are to be obtained from treatment.

To remove the acne pustules and leave the seborrhoic state unchecked is to invite relapses. A description of the formation of the acne pustule will make this clear, and will also indicate the line of treatment necessary for a cure.

### Seborrhœa and Acne.

In seborrhœa the horny layer of the epidermis is badly formed and scaly, so that the excretory ducts of the sebaceous glands, which are in connection with the lanugo hairs, become blocked with inspissated fat and loose epithelium. These glands then become distended with sebum, and in this condition they are known as comedones. This sebum forms an excellent medium for the growth of those microorganisms which are to be found in the skin. They take advantage of this nutriment placed at their disposal, flourish on it, and produce suppuration.

Acne vulgaris, therefore, is a secondary infection of comedones: it is at once obvious that to prevent acne one must remove the conditions which favour the production of comedones. Acne can never be thoroughly cured unless the seborrhœic state is treated and kept in check.

### The Method of Treatment.

The methods of treatment can be divided into three groups:—

1. Attention to the general health.
2. Removal of the pus.
3. Treatment of the seborrhœa.

Attention to the general health is of importance, for in many of these cases, owing to intestinal derangements, toxins are formed in the intestinal canal, the absorption of which must have a bad effect on the skin. In such a case a morning saline aperient should be given, the diet regulated, all indigestible articles being cut out and alcoholic drinks dispensed with. An acid mixture taken after meals can be ordered to aid digestion, or the case treated on general principles. Yeast, calcium sulphide, and cod-liver oil have their advocates. Locally the first thing to be done is to remove the pus. To effect this the skin should be softened by a couple of boric fomentations, after which the point of a sharp and narrow-bladed knife should be gently insinuated down through the orifice of each pustule till pus is reached. This is then gently squeezed out. Cutting and slicing should be avoided, as they are liable to cause scarring, which in some cases may become keloid.

The face is now massaged with the lather obtained from any good neutral (super-fatted) soap for five to ten minutes, and then rinsed clear with warm water.

This process has to be repeated again and again till all pustules have been removed. To prevent spread of suppuration some antiseptic ointment containing sulphur and mercury is necessary.

A useful one is as follows:—

| | |
|---|---|
| Precipitated sulphur ... ... ... | gr. xv. |
| Ammoniated mercury ... ... ... | gr. x. |
| Vaseline ... ... ... ... | ℥j. |

This is rubbed into the affected area, after the massage with soap and water. This line of treatment, though simple, will, if persevered in, show very good results, and will amply repay the operator and patient for all the time and trouble taken over it. The pustules are cleared away, and will leave no scars if they are operated on as suggested. When this stage has been reached all further treatment is directed against the condition of seborrhœa, and this must be kept up as long as the patient has excess of sebum on the face.

A good lather is to be formed from warm water and a neutral soap, and the face is to be massaged with it two or three times a day. Friction must be used; in this way the excess of fat is removed. In bad cases it is advisable to further this removal by means of a skin curette.

Steaming the face may be tried instead of, or in conjunction with, the washing for the same reasons.

### Local Applications.

Local applications can now be applied, and the best ones to use are ointments containing zinc oxide and sulphur or spiritous solutions of carbolic acid (gr. v. ad ℥j.) or of precipitated sulphur and glycerine. When comedones are formed the contents of the sebaceous glands must be squeezed out with any suitable instrument.

Vaccines have now been produced, and several successful cures have been published.* They, however, have their drawbacks, for in their preparation a skilled pathologist and a laboratory are necessary, and these are not always at hand; moreover, the cost is greater. Again, though the vaccine may cure the pustular condition of acne, it will not affect the underlying basis of seborrhœa, which must be treated in order to prevent relapses. In severe and long-standing cases, however, a combination of the vaccine and local treatments may be tried; but in milder ones it is hardly necessary to go to the trouble of preparing a vaccine from the patient's pus, for one can cure the condition by the methods advocated here.

In any case, whether vaccines are used or not, the cure will not be effected in a few days, so both the doctor and his patient must be content with a slow but sure progress, and must persevere with the remedies at their disposal till the result aimed at is obtained.

* Dr. French in *British Medical Journal*, February 2, 1907.

